# Girdin Knockdown Increases Gemcitabine Chemosensitivity to Pancreatic Cancer by Modulating Autophagy

**DOI:** 10.3389/fonc.2021.618764

**Published:** 2021-03-29

**Authors:** Sheng Wang, Wei Feng, Wulin Wang, Xiaoman Ye, Hao Chen, Chunzhao Yu

**Affiliations:** ^1^Department of General Surgery, The Affiliated Suqian Hospital of Xuzhou Medical University, Suqian, China; ^2^Department of General Surgery, The Second Affiliated Hospital of Nanjing Medical University, Nanjing, China; ^3^Department of Critical Care Medicine, The Fourth Affiliated Hospital of Nanjing Medical University, Nanjing, China

**Keywords:** girdin, gemcitabine, chemosensitivity, autophagy, p62/SQSTM1

## Abstract

Chemotherapy is crucial for the treatment of pancreatic cancer (PC). Gemcitabine (GEM) as the first-line chemotherapy drug has a high resistance rate. Increasing the sensitivity of gemcitabine is currently the objectives and challenges of this study. Our previous study showed Girdin was closely related to the progression and prognosis of PC, indicating that Girdin may be associated with chemosensitivity. In the current study, we use recombinant adenovirus to specifically knockdown Girdin in PC cell lines to determine the effect of Girdin in the process of gemcitabine chemosensitivity. Autophagy is one of the pathways affecting the gemcitabine chemosensitivity in PC. Further research validated that Girdin may activate autophagy by interacting with autophagy protein p62/SQSTM1, which could enhance chemotherapy resistance to gemcitabine in PC. Down-regulation of Girdin may therefore increase gemcitabine chemosensitivity in PC. Our results reveal that Girdin acted as a negative regulator of gemcitabine chemosensitivity in PC. Increased autophagy activity caused by abnormally high Girdin expression may be one of the main factors for the reduction in chemosensitivity, which may provide new perspectives on understanding chemosensitization in PC.

## Introduction

Pancreatic cancer (PC) is one of the most malignant tumors in the digestive system, and its incidence has been increasing annually worldwide (1, 2). Early diagnosis of PC is highly difficult due to the concealed pathogenesis and rapid disease progression. Most patients show locally advanced diseases or have distant metastases at the time of diagnosis, making the treatment for PC difficult and the mortality rate especially high. At present, the median survival time of the disease is only 6 months, with a 5-year survival rate of less than 5% (3). Patients with PC still rely on comprehensive treatment, including chemotherapy to improve the prognosis. Chemotherapy is one of the important methods for the treatment of PC. Gemcitabine, a deoxycytidine analogue, is known as the first-line chemotherapy drug for PC, which prevents cell cycle progression from G1 into S phase by inhibiting DNA polymerase. Gemcitabine can help prolong the median survival time and provide benefits for the patients (4). However, the rate of resistance to gemcitabine is almost above 80%, limiting the effect of the chemotherapy against PC. Chemotherapy resistance is the main reason for treatment failure in PC. The development of chemotherapy resistance is a complex process that involves multifactors, multiple genes, and multiple mechanisms. Increasing the sensitivity of chemotherapy has been one of the hotspots in PC research (5–7). At present, existing studies have shown that changes in the related genes, tumor microenvironment, cellular signaling pathways, and regulation of autophagy are the main causes for the development of chemotherapy resistance. Still, the specific mechanism remains to be improved. Therefore, further elucidating the mechanism of chemotherapy resistance, searching for anti-resistance strategies, and enhancing the sensitivity of chemotherapy drugs are still problematic in PC.

Girdin (girders of actin filament), an actin-binding protein, has received increasing attention in recent years. It is an encoded protein first named by a Japanese scientist consisting of 1,870 amino acids (8). With an extensive study, Miyake et al. (9), found that Girdin is involved in the formation of neointima after vascular injury. Feng et al. (10), have also shown that Girdin is closely associated with the migration and invasion of gliomas, and high expression of Girdin shows lower progression-free survival. Furthermore, Mikel (11) found that Girdin is involved in the regulation of autophagy through its guanine nucleotide exchange factor. Ghosh et al. (12) followed hundreds of patients and found that high expression of Girdin is positively correlated with the risk of recurrence in colon cancer patients. Interestingly, it is found that down-regulation of Girdin can enhance the sensitivity of colon cancer to chemotherapy (13). Results in the literature indicate that Girdin may play an important role in the occurrence and development of malignant tumors. However, the role of Girdin in the development of PC remains to be studied.

Previous studies from our group showed that Girdin is overexpressed in PC, which promotes cell proliferation and inhibits the apoptosis of PC cells (14). In current study, we aimed to determine the effect of Girdin in the process of gemcitabine chemosensitivity in PC and to explore its underlying molecular mechanism, especially the correlation with autophagy. Our results could help prove Girdin is a potential therapeutic target for PC by enhancing the effects of current therapy through increased chemosensitivity in PC.

## Materials and Methods

### Cell Culture and Adenoviral Infection

Human pancreatic ductal epithelial cells (hTERT-HPNE) and human PC cell lines (AsPC-1, BxPC-3, PANC-1) were acquired from American Type Culture Collection (Manassas, VA, USA). AspC-1 cells were cultured in RPMI-1640 (Gibco; Thermo Fisher Scientific, Waltham, MA, USA) while others were cultured in DMEM (Gibco). Both growth media were supplemented with 10% fetal bovine serum (FBS) (Wisent, St. Bruno, QC, Canada), and all cells were maintained in 37°C and 5% CO2.

Recombinant adenoviruses containing the Girdin shRNA (rAd-shGirdin) or an empty vector (rAd-GFP) were synthesized using the vector pAd-U6-CMV-GFP (Shanghai Lici Biotechnology, Shanghai, China). Empty vector was used as a negative control. 293A cells (ATCC) were used for packaging the recombinant adenovirus. All the cell lines and viruses have been described in the previous study (14). The P62 siRNA sequences used for expression silencing were:

siRNA-1 (forward), 5’-GUGACGAGGAAUUGACAAUdTdT-3’ and siRNA-1 (reverse), 5’-AUUGUCAAUUCCUCGUCACdTdT−3’;

siRNA-2 (forward), 5’-GGAGUCGGAUAACUGUUCAdTdT-3’ and siRNA-2 (reverse), 5’-UGAACAGUUAUCCGACUCCdTdT-3’.

### Western Blot Analysis

Proteins were extracted by RIPA buffer supplemented with Phenylmethanesulfonyl fluoride (PMSF). Cell lysates with the same amount of total protein (30 µg) were subjected to sodium dodecyl sulphate-polyacrylamide gel electrophoresis (SDS-PAGE) and transferred to polyvinylidene fluoride (PVDF) membranes (Millipore, Billerica, MA, USA). Membranes were blocked in Tris-buffered saline containing 0.05% Tween-20 (TBST) and 5% bovine serum albumin (BSA; Sigma-Aldrich; Merck KGaA, Darmstadt, Germany) for 2 h at room temperature. After blocking, the membranes were then incubated with primary antibodies at 4°C overnight, followed by the incubation with specific secondary antibodies for 2 h at room temperature. Antibodies against caspase-3, cleaved caspase-3, LC-3, beclin-1, and P62 were purchased from Cell Signaling Technology (Beverly, MA, USA). Antibodies against Girdin, Bcl-2, and BAX were purchased from Abcam (Cambridge, UK). GAPDH, β-actin, and goat anti-rabbit IgG antibodies were acquired from Santa Cruz Biotechnology (Santa Cruz, CA, USA). Relative protein expression level was evaluated using ImageJ software.

### Cell Apoptosis Analysis by Flow Cytometry

Cells were incubated with different treatment conditions. Following the treatment, cells where then collected and washed twice with ice-cold PBS, and resuspended in 1×binding buffer (BD Pharmingen; BD Biosciences, Franklin Lakes, NJ, USA). Subsequently, 5 µl of 7-AAD (BD Pharmingen; BD Biosciences) and 5 µl of APC (BD Pharmingen; BD Biosciences) were added. The samples were then analyzed *via* a flow cytometer (BD FACS Calibur equipped with Cell Quest Pro software).

### Co-Immunoprecipitation Analysis

Protein lysates (1000 μg) were prepared from cultured cells using NP40. Immunocomplex pull-down was achieved *via* overnight incubation of protein lysates with relevant antibodies bound to Glutathione Sepharose beads (GE Healthcare, USA) at 4°C. After careful washing, 2×SDS loading buffer was added and the samples boiled at 95°C for 10 min to allow for protein degeneration. Co-immunoprecipitated proteins were then subjected to western blotting as described above.

### Immunofluorescence Staining

Paraformaldehyde (4%) was used to fix pre-treated cells overnight at 4°C. Fixed cells were then incubated in PBS containing 0.1% Triton and 5 mg/mL BSA. Cells were then blocked with 5% BSA for 30 min at 37°C. Afterwards, cells were stained with antibodies against P62 (1:100; CST, Beverly, MA, USA) and Girdin (1:100; Abcam Cambridge, UK) overnight at 4°C, and subsequently incubated with a fluorescent dye-conjugated goat anti-rabbit IgG (Thermo Fisher Scientific) for 30 min at 37°C. Nuclei were stained with DAPI (Sigma-Aldrich; Merck KGaA, Darmstadt, Germany). Images were captured using a confocal laser scanning microscope (Olympus Corp.). Anti-LC3 purchased from CST (1:100) was used in another immunofluorescence staining.

### Mass Spectrometry

Cell lysates were immunoprecipitated with anti-Girdin antibody. Protein digestion, labeling, mass spectrometry data acquisition, and identification were completed in the analysis center of Nanjing Medical University. The labeled peptides were analyzed on a LTQ-Orbitrap instrument (Thermo Fisher Scientific) connected to a Nano ACQUITY UPLC system *via* a nanospray source. The LC-MS/MS was operated in the positive ion model as described previously. The MS/MS spectra acquired from precursor ions were submitted to Maxquant (version 1.2.2.5).

### Tumor Xenograft Model

Fifteen female nu/nu mice at the age of 6 weeks (Beijing Vital River Laboratory Animal Technology Co. Ltd., Beijing, China) were housed in sterile cages by conventional feeding. The left flank of each mouse was subcutaneously injected with BxPC-3 cells (5x10^6^ cells/100 µL PBS) to establish the PC xenograft model. Cells were infected with rAd-shGirdin prior to subcutaneous inoculation. After five days, the mice were randomly divided into different groups for different sets of experiments. Gemcitabine was intraperitoneally injected at a dose of 100mg/kg three times per week for three weeks. Tumor volumes were observed every three days and were calculated using the formula (A × B^2^)/2, where A and B are the long and short dimensions, respectively. Mice were sacrificed by cervical dislocation on day 21 and the weights and volumes of the tumors were measured. Half of the tumor specimens were fixed with 4% paraformaldehyde and the other half was cryopreserved at -80°C. All animals received humane care, and all experiments were performed according to the guidelines outlined in the Guide for the Care and Use of Laboratory Animals. All of our experiments were reviewed and approved by the Animal Ethics and Welfare Committee with approval no. IACUC-1601161.

### Statistical Analysis of Data

All of the experiments were carried out at least three independent times. All statistical analyses were performed using GraphPad Prism 5.0 software (GraphPad Software, Inc., La Jolla, CA, USA). All data are expressed as mean ± SEM. Data from each group were statistically analyzed using a two-tailed Student’s *t*-test. Differences with P < 0.05 were considered statistically significant. P-values shown in the figures are labeled as *P < 0.05, **P < 0.01, and ***P < 0.001.

## Results

### Girdin Expression Is Positively Correlated With Gemcitabine Chemosensitivity in PCs

**Figure 1:**
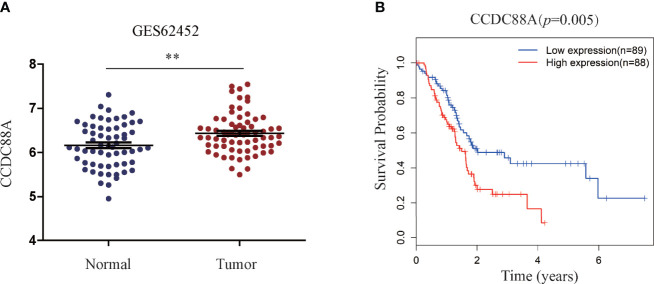
Statistical analysis of the correlation between Girdin expression and pancreatic cancer through databases. **(A)** Girdin expression level in PC tissue was significantly higher than that seen in the Normal tissue, data from GEO database, numbered GES62452. **(B)** Overall survival (OS) was compared between patients with low and high expression Girdin, data from TCGA database. **P < 0.01.

According to our previous results from tissue microarray analysis, Girdin was abnormally highly expressed in PC, and may be related to the pathological classification of PC (14). However, the microarray study did not include the prognostic relationship analysis. We therefore queried the public databases to determine whether Girdin is associated with patient prognosis. We first analyzed the gene expression data from the GEO database, accession number GES62452. We found that the expression of Girdin in tumor tissues was significantly higher than in normal tissues (P < 0.05) (**Figure 1A**). Analysis of the data from The Cancer Genome Atlas (TCGA) database showed that among the 177 patients with PC, patients with Girdin overexpression had significantly lower overall survival than the patients with relatively low levels of Girdin expression (**Figure 1B**).

**Figure 2:**
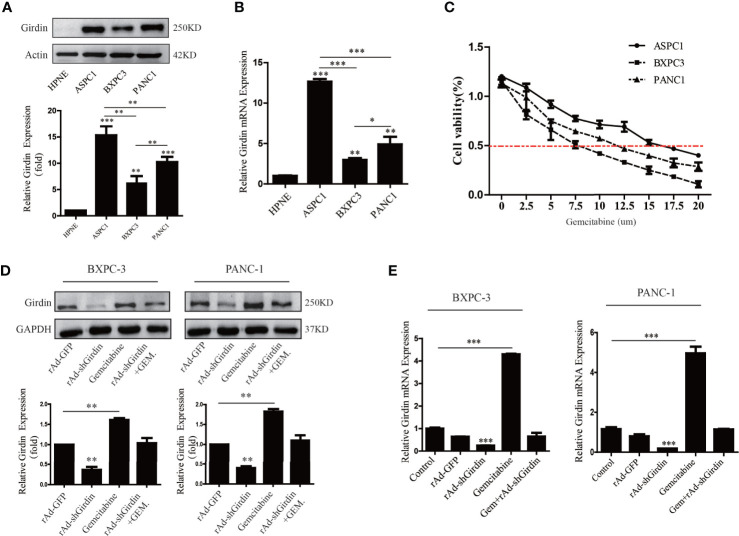
Girdin expression is positively correlated with gemcitabine chemosensitivity in pancreatic cancers. **(A, B)** Western blots and qRT-PCR were performed to analysis of Girdin protein levels in PC cell lines (Aspc-1, Bxpc-3 and Panc-1) infected with rAd-shGirdin. Bars represent the SEM. **(C)** The optimal concentration of gemcitabine in different pancreatic cancer cell lines was clarified by MTT assay. **(D, E)** Western blots and qRT-PCR were performed to evaluate the variation of Girdin level after stimulate with gemcitabine. The empty rAd-GFP vector was constructed as an experimental control. The data are representative of three independent experiments and are expressed as means ± SEM; *P < 0.05, **P < 0.01, ***P < 0.001.

One of the reasons for the poor prognosis of PC is the decreased chemotherapy sensitivity, which led us to suspect that abnormal overexpression of Girdin may affect the sensitivity to chemotherapy in PC. To further verify this conjecture, the levels of mRNA and protein expression of Girdin were analyzed in the PC cell lines AsPC-1, PANC1, and BxPC3 by qRT-PCR and western blotting, respectively (**Figures 2A, B**). The pancreatic ductal epithelial cell line HPNE was used as a contrasting non-cancer control. Both Girdin gene and protein expressions were significantly increased in PC cell lines when compared to HPNE. The highest level of Girdin protein expression was observed in AsPC-1 cells, which also display the greatest gemcitabine tolerance, with an IC50 of 15 uM. The lowest expression of Girdin protein was found in BxPC3 cells, which were the least tolerance to gemcitabine, with an IC50 of 7.5 uM (**Figure 2C**). Next, we treated PANC1 and BxPC3 cells with the corresponding IC50 concentration of gemcitabine and analyzed the expression of Girdin with western blotting and qRT-PCR. As shown in **Figures 2D** and **2E**, Girdin expression was significantly increased at protein and mRNA levels following exposure to gemcitabine. Moreover, The BXPC-3 and PANC-1 cells were successfully infected with the recombinant adenoviruses, and Girdin expression was silenced. Hence, we speculated that the level of Girdin may be correlated with the sensitivity to gemcitabine in human PC cells.

### Down-Regulation of Girdin Increased Chemosensitivity to Gemcitabine in PC

**Figure 3:**
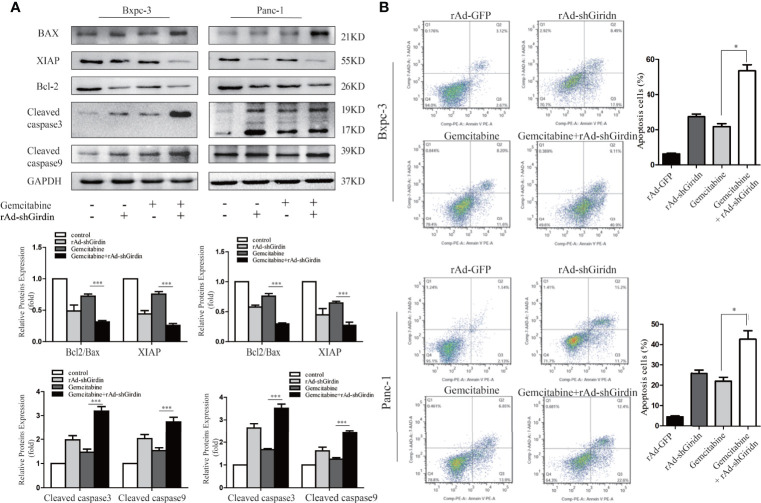
Down-regulation of Girdin increase chemosensitivity of gemcitabine in pancreatic cancers. **(A)** Apoptosis related proteins were detected by western blotting after cells were treated with single rAd-shGirdin or gemcitabine treatment, and combination of the two. The combination treatment could induce apoptosis through down-regulating the Bcl-2/Bax ratio, XIAP and activating caspase-3 and caspase-9. **(B)** Cell apoptosis was determined by flow cytometry. The combination of rAd-shGirdin and gemcitabine could induce significant apoptosis compared with the cells treated with gemcitabine alone in pancreatic cancer. Each experiment was performed three times and all data were expressed as mean ± SEM; *P < 0.05, **P < 0.01, and ***P < 0.001.

In order to further explore the relationship between Girdin and the chemosensitivity to gemcitabine chemotherapy, recombinant adenovirus of Girdin knockdown was constructed (rAd-shGirdin), while the empty rAd-GFP vector was used as an experimental control. Previous experiments have verified the effectiveness of this recombinant adenovirus. In the next experiment, we measured the expression levels of apoptosis-related proteins XIAP, Bcl-2, BAX, cleaved caspase-3, and cleaved caspase-9 by western blotting (**Figure 3A**). It was found that the levels of XIAP and Bcl-2/BAX were reduced while cleaved caspase-3 and -9 were up-regulated after a single transfection with rAd-shGirdin or gemcitabine treatment. When rAd-shGirdin infection and gemcitabine treatment were combined, we found the changes of apoptosis related proteins were more pronounced compared to the single-treatment and control groups (P<0.05), indicating there was increased apoptosis during the combined treatment. Thus, we hypothesized that Girdin knockdown may increase cellular sensitivity to gemcitabine. To further confirm our idea, the flow cytometric apoptosis experiments were performed. As shown in **Figure 3**, after treatment with an optimal concentration of gemcitabine in the rAd-shGirdin transfected PANC1 and BxPC3 cells, the combination of rAd-shGirdin and gemcitabine had a dramatically increased rate of cell death. Through the above experiments, we confirmed that down-regulation of Girdin can induce cellular apoptosis and increase the chemosensitivity to gemcitabine.

### Girdin Interacts With Autophagy Protein P62/SQSTM1 in PC Cells

**Figure 4:**
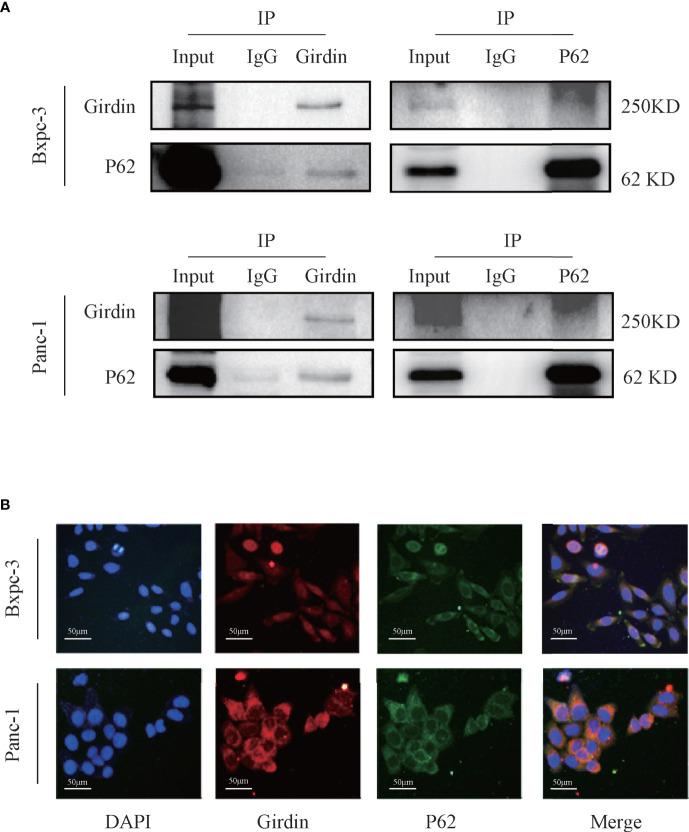
Girdin interacts with autophagy protein P62/SQSTM1 in pancreatic cancer cells. **(A)** The interaction between Girdin and P62 was detected in the co-IP analysis Bxpc-3 and Panc-1 cells. The binding signals of P62 with Girdin were also confirmed using anti-Girdin antibodies in IPs, and subsequent blotting with P62 antibodies. **(B)** The expressions of Girdin and P62 were endogenous and immunofluorescence staining images showed a significant physically interacted with each other in the cytoplasm. Red represents Girdin, green represents P62, and the nuclei were labelled with DAPI.

Girdin knockdown can increase the gemcitabine chemosensitivity in PC cells. However, its molecular mechanism remains to be further explored. In order to investigate this question, we conducted mass spectrometric analysis experiments (**Table 1**). Many of the proteins were detected that may be related to Girdin. We noticed that among the identified proteins, P62/SQSTM1 is closely related to the cellular autophagy pathway. Subsequently, we verified that Girdin and P62 could interact as shown by co-immunoprecipitation (**Figure 4A**). Further immunofluorescence experiments confirmed that Girdin physically interacted with P62 in the PC cells (**Figure 4B**), which further proves the correlation between Girdin and P62/SQSTM1.

**Table 1:** Analysis of protein interaction about Girdin in MS.

Function	Gene	Protein	ScrambleTSC^a^	Girdin^OE^TSC^b^	Value
protein folding	FKBP4	Peptidyl-prolyl cis-trans isomerase FKBP4	+	–	13
	CCT8	T-complex protein 1 subunit theta	–	+	18
	CANX	Calnexin	+	+	10
	TCP1	T-complex protein 1 subunit alpha	+	+	10
	P4HB	prolyl 4-hydroxylase subunit beta	+	+	19
***g***lycolytic process	ALDOA	Fructose-bisphosphate aldolase	+	+	12
	ENO1	Alpha-enolase	+	**-**	13
	PKM	Pyruvate kinase PKM	+	+	14
	PGK1	Phosphoglycerate kinase 1	–	+	14
	GPI	Glucose-6-phosphate isomerase (Fragment)	+	–	11
	TPI1	Isoform 2 of Triosephosphate isomerase	+	+	13
	LDHA	L-lactate dehydrogenase A chain	+	+	14
	LDHB	L-lactate dehydrogenase B chain	+	–	12
***actin cytoskeleton organization***	EZR	Ezrin	+	+	10
	MYH9	Myosin-9	–	+	99
	FLNB	Isoform 8 of Filamin-B	–	+	61
	LIMA1	Isoform 4 of LIM domain and actin-binding protein 1	–	+	27
	PLEC	Isoform 4 of Plectin	–	+	142
	CALD1	Caldesmon	–	–	14
	DBN1	Drebrin	–	+	12
	CAPZB	Isoform 2 of F-actin-capping protein subunit beta	+	+	10
	VIM	Vimentin	–	+	32
	ACTN4	Alpha-actinin-4	+	+	17
***regulation of cell death***	SQSTM1	Sequestosome-1	+	+	13
	RPS3	40S ribosomal protein S3	+	–	10
	FHL2	Four and a half LIM domains protein 2	+	+	13
	HSP90AB1	Heat shock protein HSP 90-beta	+	+	19
	HSP90B1	Endoplasmin	+	+	14
	HSPA5	78 kDa glucose-regulated protein	+	–	23
	YWHAE	14-3-3 protein epsilon	+	+	11
	HNRNPK	Isoform 3 of Heterogeneous nuclear ribonucleoprotein K	+	–	13
***translational elongation***	TUFM	Elongation factor Tu, mitochondrial	+	+	10
	RPL7	60S ribosomal protein L7	+	+	10
	EEF2	Elongation factor 2	+	+	24
***nucleotide biosynthetic process***	MTHFD1	C-1-tetrahydrofolate synthase, cytoplasmic	–	+	16
	ATP1A1	Isoform 4 of Sodium/potassium-transporting ATPase subunit alpha-1	+	–	13
***protein complex biogenesis***	JUP	Junction plakoglobin	+	–	10
	RRM1	Ribonucleoside-diphosphate reductase large subunit	–	–	11
	FLNA	Filamin-A	+	+	68
	HSPD1	60 kDa heat shock protein, mitochondrial	+	+	18
***intracellular transport***	MYO1E	Unconventional myosin-Ie	–	–	27
	HSP90AA1	Heat shock protein HSP 90-alpha	+	+	15
	CLTC	Clathrin heavy chain	+	+	19
	MYH10	Myosin-10	–	–	20
***blood vessel development***	NCL	Nucleolin	+	+	15
	ANXA2	Annexin A2	–	+	13
***protein targeting***	HSPA9	Stress-70 protein, mitochondrial	–	+	22
	SPTBN1	Spectrin beta chain, non-erythrocytic 1	–	+	44
	AKAP12	Isoform 3 of A-kinase anchor protein 12	+	–	16

Scramble Bxpc3 cells transfected with Scramble Lentivirus.

Girdin^OE^Bxpc3 cells transfected with Girdin Lentivirus.

TSC, total spectral counts.

### Down-regulation of Girdin reduces the autophagy activity of PC cells

**Figure 5:**
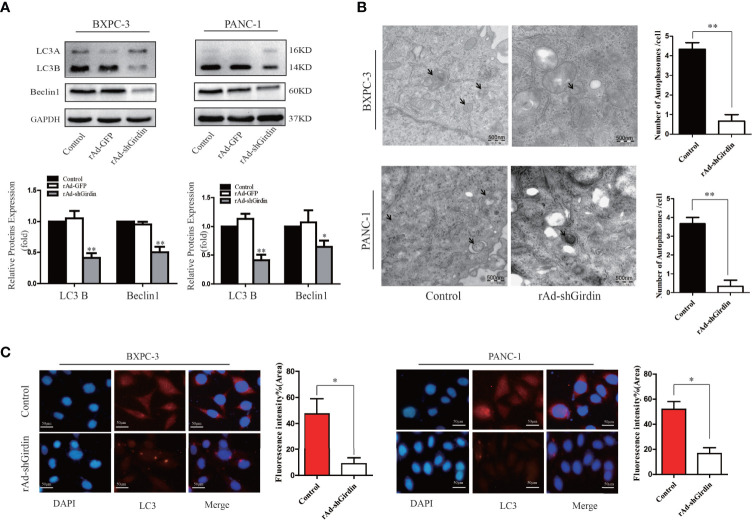
Down-regulation of Girdin reduce the autophagy activity of pancreatic cancer cells. **(A)** Autophagy related proteins LC3 and Beclin-1 were detected by western blotting after cells were transfected with rAd-shGirdin. Bars represent the SEM. *P < 0.05, **P < 0.01. **(B)** Autophagosomes were observed by transmission electron microscopy in Bxpc-3 cells and Panc-1 cells after knockdown of Girdin. Cells were cultured with serum-free DMEM. **(C)** Immunofluorescence staining images showed that the LC3 protein level was decreased after infected with rAd-shGirdin.

Due to the interaction between Girdin and P62, we next explored the effect of Girdin on cellular autophagy activity. First, we verified the effects of Girdin on autophagy-related proteins LC3 and Beclin1 by western blotting. It was found that comparing to the control group, the levels of LC3B and Beclin1 clearly decreased after down-regulating Girdin (**Figure 5A**), suggesting that knockdown of Girdin can reduce the autophagy activity in PC cells. Next, we studied the effects on the PC cells following starvation in serum-free growth conditions with or without rAd-shGirdin infection by projection electron microscopy. It was discovered that when Girdin was down-regulated, fewer autophagosomes were observed (**Figure 5B**). Immunofluorescence analysis was then performed to detect the level of LC3. Cells were starved to induce autophagy in serum-free conditions for 24 h. It can be seen that when Girdin was down-regulated, the fluorescence level of LC3 decreased, suggesting that the autophagy activity was inhibited (**Figure 5C**). The above experiments have shown that down-regulation of Girdin can reduce the autophagy activity of PC cells.

### Decreased Autophagy Activity Can Increase Gemcitabine Chemosensitivity in PC Cells

**Figure 6:**
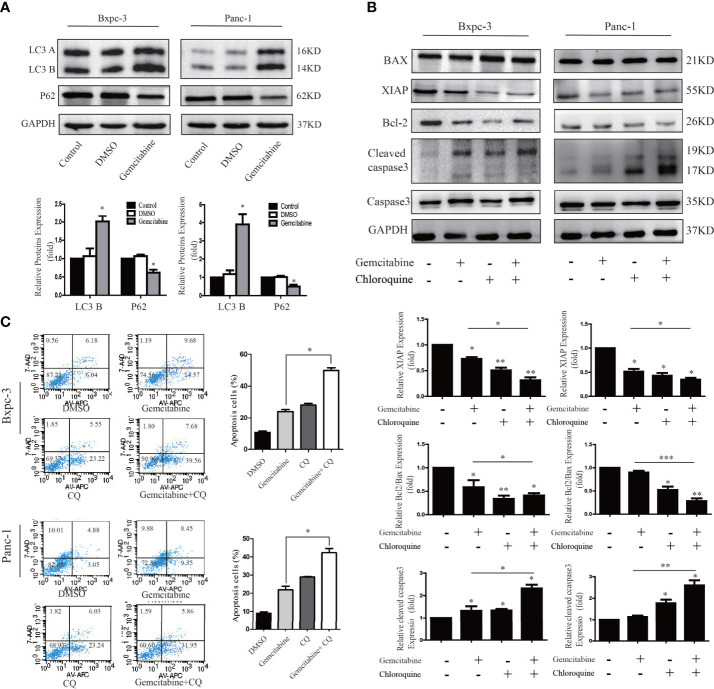
Decreased autophagy activity can increase gemcitabine chemosensitivity to pancreatic cancer cells. **(A)** Autophagy related proteins LC3 and P62 were detected by western blotting after cells were treated with gemcitabine. **(B)** Apoptosis related proteins were detected by western blotting after cells were treated with single Chloroquine (CQ) or gemcitabine, and combination of the two. The combination treatment could down-regulate the Bcl-2/Bax ratio, XIAP and activate caspase-3. **(C)** Flow cytometry was performed using Bxpc-3 and Panc-1 cells treated with gemcitabine+CQ, which significantly induced apoptosis compared with single usage in pancreatic cancer cells.

**Figure 7:**
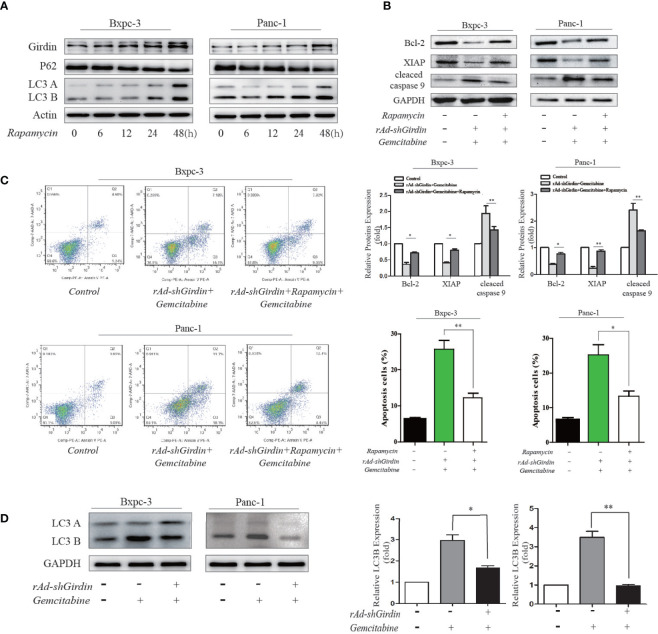
Rapamycin-activated autophagy reversed the GEM chemosensitivity caused by the downregulation of Girdin. **(A)** Girdin, P62 and LC3 were detected by western blotting after cells were induced with rapamycin. Time for 0, 6, 12, 24, 48 hours after rapamycin was administered. **(B)** Apoptosis related proteins were detected by western blotting after cells were treated with rAd-shGirdin and GEM, and with or without rapamycin applied. The combination treatment could up-regulate the Bcl-2, XIAP and down-regulate caspase-9, while compared with the group without rapamycin. **(C)** Apoptosis of BxPC−3 and PANC−1 cells was detected by flow cytometer. Cells were infected with rAd-shGirdin+GEM, and subsequently treated with rapamycin, which significantly reduced apoptosis compared with the group without rapamycin in pancreatic cancer cells. **(D)** Western blots of BxPC-3 and PANC-1 cells were performed. Levels of the LC3B proteins were decreased in the rAd-shGirdin group. Gemcitabine was used at half the previous dose to reduce cell necrosis caused by the combination of the two drugs. Each experiment was performed 3 independent times, and all data are expressed as means ± SEM; *P < 0.05, **P < 0.01.

We attempted to probe whether the level of autophagy correlates with gemcitabine chemosensitivity in the PC cells. As shown in **Figure 6A**, we treated PANC1 and BXPC3 cells with gemcitabine and detected the expression of autophagy-related proteins with western blotting. It was found that in comparison with the control group, the level of LC3B increased while the level of P62 was decreased. These results suggest that the sensitivity to gemcitabine may be related to autophagy levels. Interestingly, after Girdin was down-regulated, the autophagy activity caused by GEM was decreased (**Figure 7D**). Chloroquine (CQ) is currently an effective and commonly used autophagy inhibitor and has been widely used in many research studies (15, 16). Our study found that combined treatment with CQ and GEM significantly increased the levels of cleaved caspase-3 (P < 0.05) while the XIAP and Bcl2/Bax expression levels were significantly lower (P < 0.05) when compared with the CQ or GEM treatment alone (**Figure 6B**). Flow cytometric analysis found that when cells were simultaneously treated with CQ and GEM, the number of apoptotic cells was increased compared to single-agent exposure (**Figure 6C**). These results show that when the autophagy activity of PC was inhibited, treatment with GEM can induce higher levels of cellular apoptosis, which also means the chemosensitivity to GEM by PC cells was enhanced.

To further demonstrate its relevance, we conducted the following experiments. Rapamycin has been used as a stable autophagy activator in many studies (17). We used rapamycin to induce autophagy in the PC cell lines. Compared to the 0 h of treatment, rapamycin significantly increased the level of LC3B at 48 h along with an increase in the expression of Girdin. These results indicate that autophagy is activated after 48 hours of induction by rapamycin (**Figure 7A**). In the following experiments, we used rapamycin to induced autophagy after the cells were treated with both rAd-shGirdin and GEM combined. It was found that cleaved caspase-9 levels were lower while the XIAP and Bcl2 expression levels were higher when compared to the group without rapamycin treatment, and the differences was statistically significant (P < 0.05) (**Figure 7B**). Furthermore, a flow cytometry was carried out and to verify that the number of apoptotic cells induced by rapamycin during the combined treatment with rAd-shGirdin and GEM was reduced, indicating that rapamycin-activated autophagy reversed the GEM chemosensitivity during the downregulation of Girdin (**Figure 7C**). These results suggest that Girdin may affect the chemotherapy sensitivity in PC by regulating autophagy.

Through the above experiments, we confirmed that Girdin is closely related to the gemcitabine chemosensitivity, and it can also affect the autophagy activity of PC cells. Suppression of cellular autophagy can increase the sensitivity to gemcitabine chemotherapy in PC. Rapamycin-activated autophagy reversed the GEM chemosensitivity caused by the knockdown of Girdin. It is plausible to assume that Girdin influences the autophagy activity and regulates the GEM chemosensitivity in PC cells.

### Girdin Down-Regulation Suppresses PC Growth and Enhances Gemcitabine Chemosensitivity in a Xenograft Model

**Figure 8:**
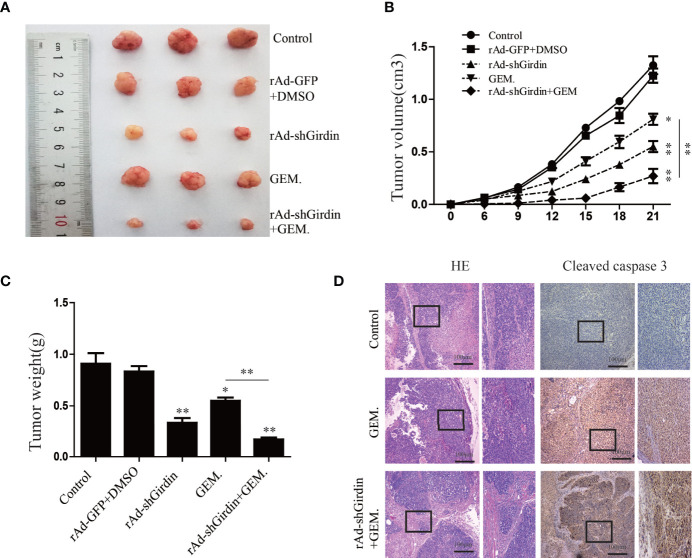
Girdin down-regulating suppresses pancreatic cancer growth and enhances gemcitabine chemosensitivity in a xenograft model. **(A)** Representative data from xenograft tumors in mice in the different groups. **(B, C)** Statistical analysis of tumour volumes and weights in the different groups (n = 4). **(D)** Images of immunohistochemical staining using antibodies against cleaved caspase-3.

We subcutaneously injected nude mice with rAd-Girdin-infected BxPC-3 cells to further confirm the effects of Girdin on PC *in vivo*. It was shown that while the expression of Girdin was knocked down the tumor growth was inhibited in the xenograft mouse model as expected. GEM was intraperitoneally injected into the mice and shown to suppress tumor growth irrespective of body weight or tumor volume. Moreover, the tumor-inhibiting capacity of GEM was further increased when rAd-shGirdin and GEM were administered concurrently (**Figures 8A–C**). Subsequent immunohistochemical staining using antibodies against cleaved caspase-3 revealed that combined treatment with GEM and rAd-shGirdin further enhanced the induction of apoptosis in tissues from xenograft tumors (**Figure 8D**), further suggesting that the down-regulation of Girdin increases the chemosensitivity to gemcitabine in PC.

## Discussion

PC is one of the most common causes of cancer-related deaths in humans, with a very high mortality rate worldwide. It was reported that adjuvant chemotherapy for postoperative patients can significantly reduce the recurrence rate of PC (18, 19). Gemcitabine is commonly used as the first-line chemotherapy drug for PC. Unfortunately, most PC patients acquire resistance to gemcitabine treatment. Ineffective chemotherapy cannot completely eradicate the cancer cells and often lead to a poor patient prognosis. Clarifying the molecular mechanism that occurs during the development of gemcitabine chemoresistance will improve the PC treatments.

Cancer chemoresistance results from a complex interplay between gene regulations. A great deal of studies have reported the main causes of chemotherapy resistance, such as changes in related genes, tumor microenvironment, novel activation of cellular pathways, autophagy, and many other factors. Our previous study has confirmed that Girdin promotes the proliferation, migration, and invasion of PC cells. Most importantly, down-regulation of Girdin could induce the apoptosis in PC cells. We also found that Girdin arrests cell cycle at G1 phase, which has similar function as gemcitabine. We therefore proposed that a combination of gemcitabine and down-regulation Girdin may have a synergistic effect that could improve the sensitivity to gemcitabine.

**Figure 9:**
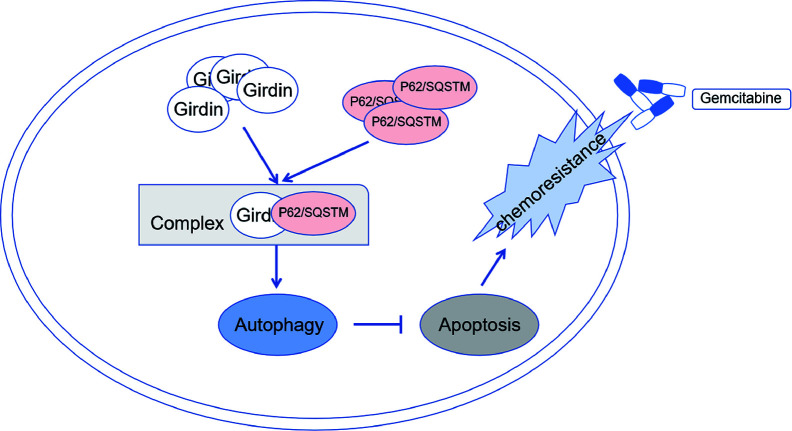
Schematic diagram showing Girdin may activate protective autophagy of pancreatic cancer cells by directly binding to p62 form as a complex, and then increase the gemcitabine chemoresistance of pancreatic cancer.

Here, we first sought to examine the expression of Girdin in PC and its impact on the prognosis. GEO and TCGA databases were interrogated, from which we found that Girdin is expressed at high levels in PC tissues and cells, and the expression correlates with the OS of PC. Next, *in vitro* experiments found that the higher expression level of Girdin in each PC cell line, the lower sensitivity of the cell line to gemcitabine, which suggested that Girdin may be associated with chemosensitivity in PC. Further analysis found that after down-regulating Girdin, the chemotherapy sensitivity to gemcitabine was increased in PC cell lines, resulting in a higher rate of apoptosis. Mass spectrometry analysis experiments were then performed, from which an autophagy-related protein P62/SQSTM1 was identified. P62/SQSTM1 is an important selective autophagy adaptor protein, and its relationship with autophagy is bidirectional (20, 21). In one hand, the intracellular p62 level is strictly regulated by autophagy activity. On the other hand, p62 can also negatively regulate the autophagy activity of cells by activating the mammalian target of rapamycin complex 1 (mTORC1) signaling pathway. The expression level of p62/SQSTM1 has also been used as a marker for autophagy activity in other studies (22). At the same time, we found that when Girdin was down-regulated, PC cell autophagy activity, which is often known as one of the possible causes of chemotherapy resistance, was significantly decreased. Autophagy is an evolutionarily conserved self-defense mechanism, which sequesters and degrades proteins and organelles. Usually, autophagy can dispose damaged mitochondria and reduce the incidence of cancer (23). Once tumor has occurred, autophagy may maintain cell survival in response to hypoxia and nutrient limitation (24). Tumor cells can take advantage of autophagy, resist the damage and the apoptosis process induced by chemotherapeutic drugs (25, 26). Many studies have described the relationship between autophagy and chemoresistance in cancers, which found that the suppression of autophagy activity can often enhance the chemosensitivity. The development of many different cancers is accompanied by a high level of autophagy, including PC (27–29). Perera et al. (30) discovered that cellular stress caused by autophagy can alter the cell metabolism, which in turn promotes the development of PC. Therefore, we hypothesized that Girdin may activate the protective autophagy in PC cells by directly binding to p62/SQSTM1, increasing the gemcitabine chemoresistance in PC. Down-regulation of Girdin can increase the sensitivity to gemcitabine chemotherapy in PC (**Figure 9**). Next, we further confirmed that the autophagy activity of PC cells is directly related to its chemosensitivity. Chloroquine was employed as an inhibitor that can reduce autophagy activity, and simultaneously increases chemosensitivity. Therefore, Girdin may affect the chemosensitivity of PC cells through the autophagy pathway. The author further conducted western blotting to verify the regulatory correlation between Girdin and P62. Interestingly, it was found that P62 did not significantly changed after Girdin was down-regulated. Similarly, after P62 was down-regulated, Girdin was not significantly changed (**Supplemental Figures S1A, B**) Therefore, we suspected that Girdin and P62 may form a complex that results in the promotion of protein function to affect autophagy activity in PC cells. Next, it was further verified in mouse tumor-bearing experiments that down-regulating Girdin could increase gemcitabine sensitivity in PC, which was consistent with the results from *in vitro* experiments.

Admittedly, Girdin has multiple functional domains, including AKT phosphorylation sites which involved in the PI3K/AKT signaling pathway. The functional domain GEF of Girdin is related to Gα-interacting vesicle-associated domains associated with the hook-related proteins. Girdin protein function is diverse. Some scholars (11) have shown that in HeLa cells, Girdin can inhibit autophagy by activating the G protein. However, there are many major activators for the regulation of autophagy, and many different factors and conditions maintain the balance between promotion and inhibition of autophagy. The underlying molecular mechanism describing the relationship between Girdin and autophagy needs to be further studied. In addition, some researchers have confirmed that Girdin is also closely linked to the activation of the PI3K/AKT signaling pathway (31). The PI3K/AKT signaling pathway controls cell growth and death, and to some extent, also changes the sensitivity to chemotherapy (32). While it may not be the only mechanism that Girdin influences gemcitabine chemosensitivity in PC by modulating autophagy, this protein truly plays a vital role in the regulation of chemosensitivity.

In conclusion, we found that the abnormally high expression of Girdin affects the prognosis of PC and the chemosensitivity to gemcitabine. Down regulation of Girdin can increase the chemosensitivity in PC cells. In addition, Girdin maintains the delicate equilibrium of autophagy in PC cells *via* P62. Increased autophagy activity caused by abnormally high Girdin expression may be one of the main reasons for the decreased sensitivity of PC cells to gemcitabine chemotherapy. Our results suggest that a combination of Girdin-targeted therapy and gemcitabine chemotherapy is potentially useful as a novel therapeutic approach for the treatment of resistant PC in the clinical setting. Its effectiveness and detailed molecular mechanisms remain to be further explored.

## Data Availability Statement

The original contributions presented in the study are included in the article/**Supplementary Material**. Further inquiries can be directed to the corresponding authors.

## Ethics Statement

The animal study was reviewed and approved by Animal Ethics and Welfare Committee with approval no. IACUC-1601161.

## Author Contributions

All authors contributed to the article and approved the submitted version. SW and WF contributed equally to this paper as the main completer of the experiments and article. WW designed and carried out some experiments. XY helped with animal experiment and cell culture. HC was responsible for the statistical analysis of their results. CY provided funds and designed ideas.

## Funding

This work was funded by grants from the National Natural Science Foundation of China (nos. 30972910 and 81172269) and the key research and development Foundation of Suqian, China (S201809).

## Conflict of Interest

The authors declare that the research was conducted in the absence of any commercial or financial relationships that could be construed as a potential conflict of interest.

## Supplementary Material

Supplementary Figure 1 Regulatory correlation between Girdin and P62. **(A)** Western blots were performed to analysis of Girdin protein levels after transfected with P62-siRNA. Bars represent the SEM. **(B)** Western blots were performed to analysis of P62 protein levels after infected with rAd-shGirdin. The data are representative of three independent experiments. Bars represent the SEM. ***P < 0.001.
