# Supplementary figures and images for: Girdin Knockdown Increases Gemcitabine Chemosensitivity to Pancreatic Cancer by Modulating Autophagy
Source: Front Oncol. 2021 Mar 29;11:618764. doi: 10.3389/fonc.2021.618764 (PMC8039524; doi:10.3389/fonc.2021.618764)

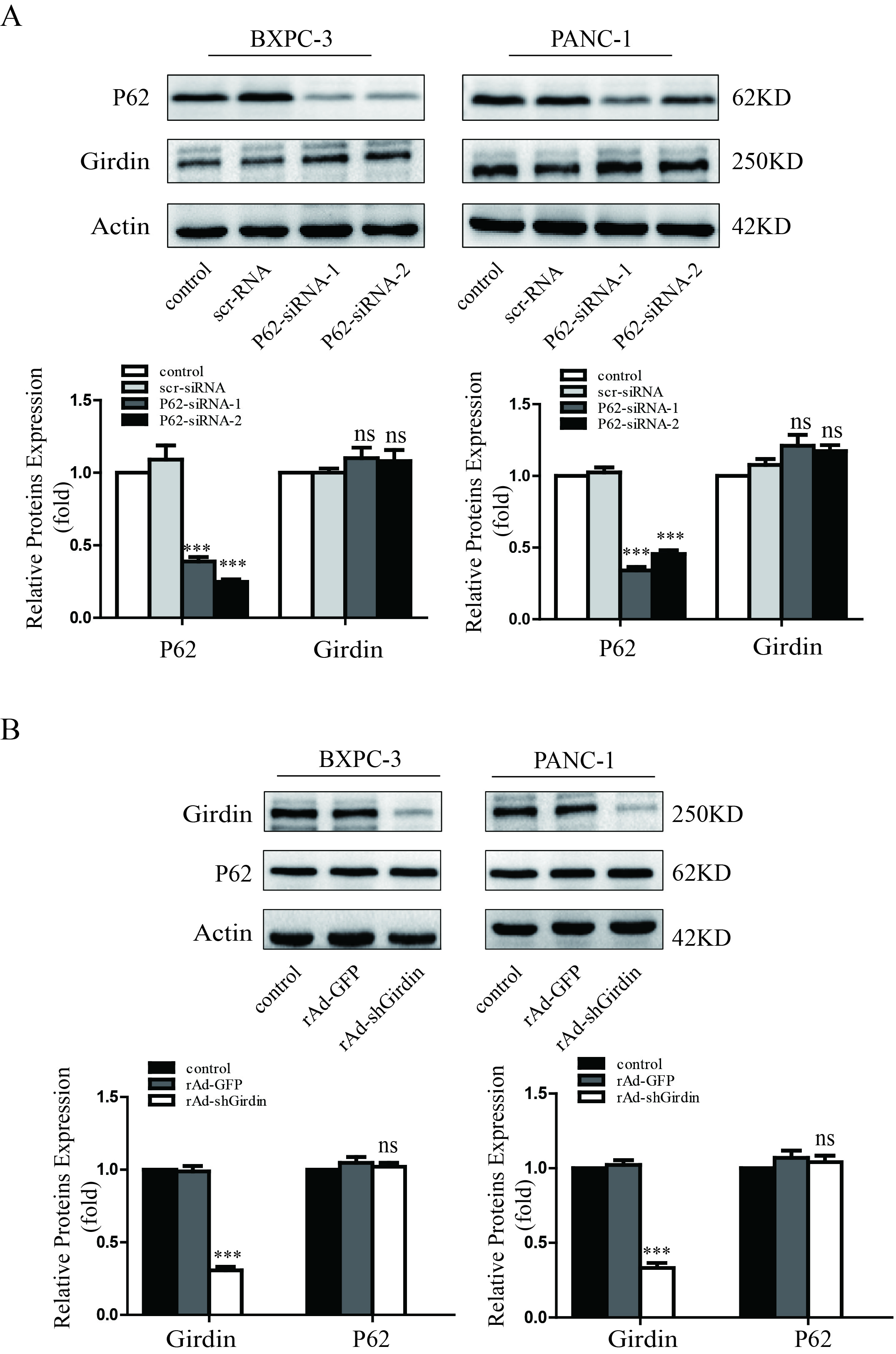

Supplement: Supplementary Figure 1 — Regulatory correlation between Girdin and P62. (A) Western blots were performed to analysis of Girdin protein levels after transfected with P62-siRNA. Bars represent the SEM. (B) Western blots were performed to analysis of P62 protein levels after infected with rAd-shGirdin. The data are representative of three independent experiments. Bars represent the SEM. ***P < 0.001. [file Image_1.jpeg]
